# Quality of intrapartum and newborn care in public healthcare facilities of Wolkite town, Central Ethiopia: facility-based cross-sectional study

**DOI:** 10.3389/fgwh.2025.1444184

**Published:** 2025-03-18

**Authors:** Berhanu Semra Mulat, Amare Zewdie, Abebaw Wasie Kasahun, Molla Gashu, Adiam Nega, Tamirat Melis

**Affiliations:** ^1^ Department of Public Health, College of Medicine and Health Sciences, Wolkite University, Wolkite, Ethiopia; ^2^ Department of Heath System and Policy, School of Public Health, College of Health Sciences, Addis Ababa University, Addis Ababa, Ethiopia

**Keywords:** quality, intrapartum, newborn, care, Ethiopia

## Abstract

**Background:**

Quality of intrapartum and newborn care is increasingly recognized internationally as a critical aspect of the unfinished maternal and newborn health agenda. Although the world has made significant progress in reducing maternal and newborn mortality, there are still far too many preventable and treatable maternal and newborn deaths globally. Poor-quality intrapartum and newborn care along with inadequate access to basic maternal and newborn healthcare services has contributed to high maternal and child mortality in low- and middle-income countries. However, there is not enough evidence describing the status quality of intrapartum and newborn care in Ethiopia, specifically in the study area. Thus, this study aims to assess the quality of intrapartum and newborn care in public health facilities of Wolkite town, Central Ethiopia.

**Methods:**

A facility-based cross-sectional study design was conducted from March to April 2023 among five public health facilities, and observation of health service provision was employed among 185 mothers. A consecutive random sampling method was applied. Data were collected through document review, interview, health service provision observation, and health facility audit against the standard checklist. Quality of intrapartum and newborn care was measured using standard intrapartum and newborn care criteria. Thus, good-quality care was considered if the mother and newborn scored 75% or more of the intrapartum criteria during childbirth. Data were entered, coded, and cleaned using EpiData version 4 and exported to SPSS version 25 for analysis. Descriptive summary statistics including proportions, mean, and median were computed to describe study variables. Multivariable logistic regression analysis was performed to identify factors significantly associated with the outcome variable. Finally, adjusted odds ratios with 95% confidence intervals and *p*-values <0.05 were considered to declare the statistical significance level of a variable.

**Result:**

The study revealed that the level of good quality of intrapartum and newborn care was 35.1% and 69.7%, respectively. Input quality (AOR = 4.52; 95% CI 1.31, 14.98), health workers with 5 or more years of experience (AOR = 7.23; 95% CI 1.49, 35.84), received on job training (AOR = 5.82; 95% CI 1.91, 13.61), and friendly maternal and newborn care (AOR = 6.89; 95% CI 1.34, 35.62) were significantly associated with quality of intrapartum care.

**Conclusion:**

The quality of intrapartum care is found poor in the study area. Clients are not getting intrapartum care with state-of-the-art knowledge and current clinical best practices. Input quality, the experience of healthcare providers, friendly care, and continuous training were factors associated with the quality of intrapartum care. Improving the availability of essential inputs, enhancing the performance of healthcare providers through training, and continuous objective monitoring of the standard clinical practices are crucial to ensuring the quality of intrapartum care.

## Introduction

Intrapartum and newborn care represents the entire range of services offered to mothers and babies from the onset of labor to the period immediately after birth, which includes monitoring, managing complications, providing interventions, and ensuring a safe transition for the baby into neonatal life (1, 2). Quality of intrapartum and newborn care is increasingly recognized internationally as a critical aspect of the unfinished maternal and newborn health agenda (3). The World Health Organization recommends that every pregnant woman and newborn receives high-quality care throughout the intrapartum period, as the time around childbirth is the most critical for saving the maximum number of maternal and newborn lives (4).

Although maternal mortality has declined from 339 to 223 per 100,000 live births between 2000 and 2020 globally, there are still far too many preventable and treatable maternal and newborn deaths due to poor-quality intrapartum and newborn care in health facilities (5, 6). This number is far behind the Sustainable Development Goal (SDG) that aims to reduce maternal mortality ratio (MMR) to less than 70 per 100,000 live births and neonatal mortality to at least as low as 12 per 1,000 live births (7). Each year, more than 295,000 women die from complications of pregnancy and delivery, and an estimated 2.4 million children die within their first month of life (8), 94% of these deaths occur in low- and middle-income countries (LMIC) and sub-Saharan Africa (5, 8).

There is strong evidence showing that the highest magnitude of maternal death has happened around childbirth and the immediate postpartum period (9). Improvement in quality of care throughout the intrapartum period can have a significant impact on maternal and newborn survival and well-being (10). Enhancing the quality of intrapartum and newborn care can be achieved through ensuring access to skilled birth attendants with evidence-based practice and respectful care, creating a supportive environment, using effective clinical and non-clinical best practices, building healthcare infrastructure, and ensuring healthcare providers have optimum skills, knowledge, and positive attitude (11, 12).

The lives of over 3 million mothers and babies can be saved every year with high coverage of high-quality intrapartum and newborn care (13). According to the results of an evidence-based intervention study conducted in 2020 among 81 countries, the provision of good-quality healthcare could reduce maternal and infant mortality by 28% and stillbirths by 22% when compared to scenarios without any improvement in quality of care (10).

In sub-Saharan Africa, the quality of intrapartum care in public health facilities remains a challenge, and these problems are made worse by the health system’s struggles to meet both the demand for routine quality healthcare and the frequent need to respond to drought, conflict, and disease outbreak including COVID-19 (14). According to estimates, in low- and middle-income countries (LMIC), about six out of ten neonatal problems and 50% of maternal deaths are due to inadequate quality of intrapartum and newborn care (15). Hence, achieving high-quality, effective, safe, people-centered, timely, equitable, integrated, and efficient care is crucial to advancing women's and neonatal health outcomes (16, 17).

Despite remarkable progress in reducing the rate of maternal and newborn mortality over the last decades, Ethiopia still experiences one of the highest maternal and newborn mortality rates in the world which stood at 401 per 100,000 live births and 33 per 1,000 live births, respectively (18). According to the FMoH report, in Ethiopia in 2017, 49% of reported maternal deaths occurred after women were found at health facilities, 14% of that maternal mortality was due to a shortage of supplies and instruments, 11% due to patient management delay at the facility, 6% due to care providers error and mismanagement, and 28% due to referral delays from different facilities (19). Therefore, increasing access to and utilization of maternal care alone is insufficient to enhance maternal health outcomes (15).

A study conducted in 2021 assessed the standard of maternal and newborn care in Ethiopia, revealing that most health facilities did not comply with the national standards for maternal and newborn health (MNH) quality of care requirements (15). Only 15.6%, 9.3%, and 10.7% of health facilities met the standard care for MNH and fulfilled the expected input, process, and output maternal and neonatal healthcare quality criteria, respectively (11). In 2016, Ethiopia adopted the World Health Organization’s standard for raising the quality of maternity and newborn healthcare in health institutions and developed a health sector quality transformation that aids in national quality strategy (NQS) implementation (16, 20). There are numerous methods for consistently measuring the process of various maternal and newborn health (MNH) care (21).

According to the Donabedian model, quality is measured in terms of three major aspects, namely, the structure (materials, infrastructure, and human resources), process (adherence to standard care throughout intrapartum and postpartum periods), and outcome (maternal satisfaction with the service's accessibility and use of emergency obstetric and newborn care) (22). Ethiopia's healthcare strategy has given due attention to the quality of health services recently (23). Therefore, generating rigorous evidence on the level of intrapartum quality of care will support the implementation of the national healthcare quality strategy.

One of the major obstacles to improving the quality of intrapartum and newborn care in low- and middle-income countries (LMIC) is the absence of relevant and reliable evidence (24). Although the quality of intrapartum care is essential for further improvement of maternal and neonatal health outcomes, there is not enough evidence about the status of the quality of intrapartum and newborn care in Ethiopia specifically in the study area.

The majority of the available studies mostly focused on survey-based client satisfaction (25–27), and few studies tried to assess the quality of intrapartum care through structure, process, and outcome of quality measurement (28). However, the relationship between the quality of the input and process of the intrapartum care was not evaluated in the previous studies. Therefore, the purpose of this study is to fill the knowledge gap by objectively assessing the quality of intrapartum and newborn care through survey and service provision observation methods. Furthermore, the association between the input and process quality of intrapartum care is examined in the current study.

## Materials and methods

### Study design, period, and setting

A facility-based cross-sectional study design was conducted from March to April 2023 in the public health facilities of Wolkite town. Wolkite town is the administrative center of the Gurage Zone in Central Ethiopia and is located 155 km west of the capital city of the country, Addis Ababa. The town has three sub-cities and six kebeles (small administrative units). According to Wolkite town’s administration health office, the total population is expected to be 61,309. Out of the total population, 31,268 (51%) are females, and the rest 30,041 are males. The number of women who are in the child-bearing age group (15–49) is 13,568; from this, 2,128 are pregnant and expecting deliveries. There are three government health centers, one specialized hospital, one primary hospital, and 11 private clinics in the town. In Ethiopia including the study setting there is a medical cost exemption for maternal health services consisting of antenatal care (ANC) and intrapartum care provision. However, clients may experience the cost of transportation and other associated expenses.

#### Populations and sample

All mothers who gave birth at public health facilities of Wolkite town were considered the source population. All mothers who gave birth at public health facilities of Wolkite town during the period of data collection were included in the study, while mothers who came for elective cesarean section delivery during the period of data collection period were excluded. Moreover, all public health facilities providing maternity services were included in the study. A total of 185 laboring mothers and skilled birth attendants from five health facilities were included in the study.

Sample size calculation for laboring mothers was determined by a single proportion of a finite population with a 95% confidence interval and marginal error (*d*) of 5% and by taking a 12.6% proportion (P) of respectful maternal care (29). Adding 10% to the non-response rate, a total of 185 mothers attending obstetric care were selected for the observation and document review. By considering the assumption of delivery attendance, a consecutive random sampling method was applied in the selected health facilities. The study participants were allocated proportionally to each health facility based on the number of delivery services provided in each health facility for 3 months prior to the study period.

#### Study variables

##### Dependent variable

*Quality of intrapartum care* is a composite variable measured using 92 items taken from the WHO guidelines, national guidelines (3, 30), and other studies that are consistent with the national guidelines. The included assessment of activities during the examination of mothers at admission, care during the various stages of labor (first, second, third), and the immediate postpartum period and infection prevention practice. The response of each item was rated 1 for “yes” and 0 for “no” by giving equal weight to all items. Then, the quality of intrapartum care was measured as good process quality if they scored ≥75% from process quality domain items (31).

##### Independent variables

*Maternal-related* (age of mothers, level of education, economic status, marital status, residence, ANC follow-up, parity and mode of delivery), *physical- and human-related* (availability of skilled care provider, type of institution, infrastructures, functional diagnostic service, availability of transportation, and distance from health facilities), *interpersonal process-related* (privacy, respectful, information provision politely, support and involvement of family members), *health professional-related* (profession, sex, and experience), and *technical process-related* (adherence to established standard care).

*Input quality* was measured using 40 items adopted from the WHO standards and other studies that are consistent with the national guidelines (3). These include the availability of basic equipment, infrastructure, and essential drugs. The response of each item was rated 1 for “yes” and 0 for “no” by giving equal weight to all items. Thus, input quality was measured as good quality if they scored ≥75% of the input quality score (31).

*Quality of newborn care* was measured using 11 items adopted from the WHO standards similar to the national guidelines (3, 30). This is part of the intrapartum quality care. The response of each item was rated 1 for “yes” and 0 for “no” by giving equal weight to all items. If they scored ≥75% of the items, they are considered as receiving good quality care (31).

*Friendly mother and newborn care* was measured using nine items. The response of each item was rated 1 for “yes” and 0 for “no” by giving equal weight to all items. Good friendly care was measured if they scored ≥75% of friendly care criteria (3). This is part of the intrapartum quality care (31).

*Comprehensive emergency obstetric and newborn care (CEMONC)* is a set of key obstetrics services or signal functions for pregnant women and newborns that may experience fatal complications, such as severe bleeding, infection, prolonged or obstructed labor, eclampsia, and asphyxia in the newborn (32).

*Completeness of the partograph* was measured using 14 items. The response of each item was rated 1 for “yes” and 0 for “no” by giving equal weight to all items. Completeness was assessed if ≥80% of the 14 items were filled correctly in the partograph to be categorized as a complete partograph (31).

*Knowledge of a skilled birth attendant* (SBA) was measured using 17 items. Firstly, the responses for each item were coded as “1” if the response was correct, otherwise coded as “0.” Secondly, individual responses to each item was summed up. Finally, individuals who correctly responded ≥70% of the knowledge items were categorized as having “good knowledge”, otherwise coded as having “poor knowledge” (31).

#### Data collection tools

To assess the quality of intrapartum and newborn care, the data were collected by using observation, document review, structured interview guide, and facility audit. Data collection tools that were in line with national guidelines were adapted from different literature, and WHO standard was used to collect all the necessary data (3, 11, 31, 33). A facility inventory checklist was used to assess the input component to interview the head of the facility and experienced maternity about the availability of equipment, drugs, and supplies at the time of the data collection period to assess input quality care. An observation checklist was used to observe service provision during childbirth care starting from admission in the labor ward till the immediate postpartum period to assess the quality of intrapartum care, newborn care, and women- and newborn-friendly care. A structured interview guide was used to conduct interviews with SBAs to describe their experiences and knowledge level. A document review form was used to gather data from the mothers’ charts to assess the completeness of the partograph.

#### Data collection procedures

There were two BSc public health professionals as supervisors and seven BSc midwives as data collectors who were working in health facilities other than the study area. The data collectors were properly dressed and had an adequate number of unfilled questionnaires. Written informed consent and permission letters from the maternity ward were obtained prior to the survey. The data collectors explained the purpose of the study for both the mother and healthcare professional, and written informed consent was obtained from both before starting data collection. First, the data collector identified of availability of basic resources for the provision of intrapartum and newborn care using observation and interviewing the head of the institution, a focal person of labor and delivery at the facilities. Second, the data collector stayed in the delivery room without interfering with the care being provided to mothers and newborns and observed the entire labor, including history taking, physical examination, diagnosis, and women-friendly care (interpersonal and technical aspects). The observation covered the active phase of the first stage of labor, followed by continuous observation of the second and third stages of labor, newborn care, and the immediate postpartum period. Additionally, the document about partograph usage was reviewed. To minimize observer bias between data collectors and the Hawthorne effect, the first three observations were excluded from the data analysis.

#### Data processing and analysis

To assess the quality of intrapartum and its predictors, both descriptive and inferential statistics were used. Data were checked for completeness; then cleaned, edited, coded, and entered into EpiData version 4; and exported to Stata version 14 and SPSS version 25 for analysis. Before the data analysis, the data were screened for missing and data entry errors using the frequency distribution of the variables and observation of the entered data. Suspected errors were validated against the raw data, and necessary corrections were made. Descriptive and summary statistics were computed. Binary logistic regression was performed to identify determinants of maternal satisfaction with the quality of intrapartum care. Variables with *p*-values of ≤0.25 in the bivariate analysis outcome were selected and included in the multivariable regression analysis. Finally, adjusted odds ratios with 95% confidence intervals and *p*-values <0.05 were considered significant independent factors of maternal satisfaction with the quality of intrapartum and newborn care. Model fitness was checked using the Hosmer–Lemeshow test (*p* = 0.772) by randomly inserting some of the variables whether the measured items correctly explained the dependent variable (quality of intrapartum care).

#### Data quality assurance

To enhance the quality of the data, the data collectors and supervisors were trained for 2 days on the objective and methodology of the research and data collection approach. The questionnaires were translated into the local language Amharic to increase the response rate. Then a questionnaire developed in Amharic was back translated into English to ensure the validity of the question. Regular supervision and meeting were made daily, and any problems raised during data collection were solved immediately. Pre-testing of the questionnaire was performed on 5% of the sample in Agena primary hospital which was not included in the actual study, and the questionnaire was modified based on the result of the pretest study. To check the internal consistency of the questionnaires, Cronbach's *α* test was performed (*α* coefficients = 0.904). All the questionnaires and data were checked for completeness and accuracy before, during, and after data collection, and double data entry was used to avoid data entry errors.

#### Ethical consideration

The ethical issues were considered throughout the study by considering the basic ethical research principles. Ethical clearance was obtained from the institutional review board (IRB) of Addis Ababa University, College of Health Science, School of Public Health, and a permission letter to carry out the study was obtained from Gurage zone health bureau. All study participants were given detailed information about the aims and methods of the study prior to the interview and observation, and written informed consent was obtained to participate in the current study. Confidentiality of information and privacy of participants was assured, and a close and harmonious relationship was established.

## Results

### Sociodemographic characteristics

In this study, service provision sessions were observed for 185 mothers and healthcare professionals in charge of providing the services for these mothers. More than half of the mothers were within the age range of 25–34 years. The majority (83.9%) of respondents were Gurage by their ethnicity, and almost all 175 (94.5%) were married. Of the total mothers, 90 (48.6%) attended primary school. Regarding health professionals, more than half of the skilled birth attendants who gave the service were female 106 (57.3%), their work experience is within the range of 2–5 years 81 (43.7%), and 84 (45.4%) of deliveries were conducted by degree-level midwives (Table 1).

**Table 1:** Sociodemographic characteristics of mothers and healthcare professionals study on quality of intrapartum and newborn care in public health facility Wolkite town, Central Ethiopia, 2023 (*N* = 185).

Variables	Categories	Frequency	Percent
Maternal age	15–24	51	27.5
	25–34	125	67.5
	35–49	9	5.0
Maternal educational status	Unable to read and write	20	10.8
	Read and write	25	13.5
	Primary(1–8)	90	48.6
	Secondary (9–12)	36	19.5
	College/university	14	7.6
Maternal marital status	Married	175	94.5
	Unmarried	10	5.5
Maternal resident	Rural	87	47.0
	Urban	98	53.0
ANC follow-up for current birth	No	17	9.2
	Yes	168	90.8
Care providers’ sex	Male	79	42.7
	Female	106	57.3
Care providers’ age	21–25	42	22.7
	26–30	104	56.2
	≥31	39	21.1
Care providers’ profession	Midwife degree	84	45.4
	Midwife diploma	41	22.1
	Nurse	14	7.5
	HO	9	5.0
	Doctor	12	6.5
	Student	25	13.5
Care providers’ marital status	Unmarried	112	60.5
	Married	73	39.5
Care providers’ work experience	<2 years	34	18.4
	2–4 year	81	43.7
	≥5 years	70	37.9
Training	No	154	83.2
	Yes	31	16.8

### Availability of resources and infrastructure (input quality)

In the five health facilities, 20 focal health professionals were interviewed, and verification by observation was conducted to assess input quality. Accordingly, out of the five health facilities, only three scored ≥75% of input quality items and were considered as having a good input quality. All facilities have electric power whereas only two had a standby backup automatic generator which starts within 5 min. However, only 40% of the health facilities have an adequate water supply. Functional basic laboratory tests (blood group, HCT, WBC count, and Rh test) were available only in one health facility.

Regarding human resources, all skilled healthcare professionals working in the delivery room and included in this study did not receive refresher training on managing obstetric complications, and only one facility has at least one skilled birth attendant trained to provide neonatal resuscitation. In addition, most of the health facilities lack towels to dry and wrap babies after delivery. Only one health facility has a functional toilet with shower service. Among the studied health facilities, only one health center and two hospitals gave all BEmONC and CEmONC signal functions, respectively (Table 2).

**Table 2:** Availability of infrastructure, human resources, drugs, and supplies for quality of input dimension in public health facilities of Wolkite town, Central Ethiopia 2023 (*N* = 5).

Input quality variables	Available	
	Yes (%)	
Infrastructures indicators		
Clean water source and supply available	2	40%
Reliable electricity available	5	100%
Means of communication available at all times even if not locked	2	40%
24 h service available	5	100%
Functioning transport facilities (ambulance) available	4	80%
At least three rooms available for maternity service	5	100%
Functional refrigerator for storage of drugs and vaccines available	4	80%
Mother toilet with shower service available	1	20%
Basic equipment and supplies in the delivery room		
Functional blood pressure apparatus available	5	100%
Functional oral or axillary thermometer available	3	60%
Functional stethoscope available	2	40%
Functional fetal stethoscope available	5	100%
Functional baby weighing scales available	4	80%
Sterilizers (autoclave or dry oven) available	4	80%
Antiseptics (soap, chlorine solution, alcohol, iodine)	5	100%
Container for infection prevention (decontamination container, safety box, covered contaminated waste bin) available	5	100%
Functional movable delivery light available	2	40%
Functional vacuum extractor available	4	80%
Essential obstetric equipment (absolute minimum equipment for delivery)		
Two sterilized delivery sets (cord scissors, cord tie, two artery forceps) available	3	60%
Episiotomy set (catgut, one tissue forceps, one needle holder, one scissor/blade) available	2	40%
Consumable supplies		
IV set and cannula available	5	100%
Folly catheter available	4	80%
Blank partograph available	5	100%
HIV test kit available	4	80%
IV fluid (normal saline) available	5	100%
Emergency drugs for maternal care available		
Oxytocin drug available	5	100%
Antibiotics (at least ampicillin, gentamicin, and metronidazole) available	4	80%
Anticonvulsant drugs [at least magnesium sulfate (MgSo4)] available	4	80%
Antihypertensive drug (hydralazine) available	4	80%
Essential newborn care equipments and drugs		
Newborn resuscitation materials (at least bags and masks, mucus extractors) available	5	100%
At least two towels to dry and warp baby after delivery available	2	40%
Functional incubator (enough light or radiant warmer) available	2	40%
Vitamin K available	5	100%
Tetracycline (TTC) eye ointment available	5	100%
Functioning laboratory service		
Basic blood tests available (HGB or HCT, WBC count, ABO blood group, and Rh test)	1	20%
Malaria and HIV tests, urine analysis like protein urea available	3	60%
Human resources for maternity services available		
At least 3 midwives available at the health center or at least 13 midwives at hospital	5	100%
Skilled birth attendance (SBAs) readiness (available and properly dressed at delivery room)	3	60%
All SBAs in the facility trained to manage obstetric complication	0	0%
At least one SBA in the facility trained to manage neonatal resuscitation	1	20%

### Quality of intrapartum care

**Figure 1:**
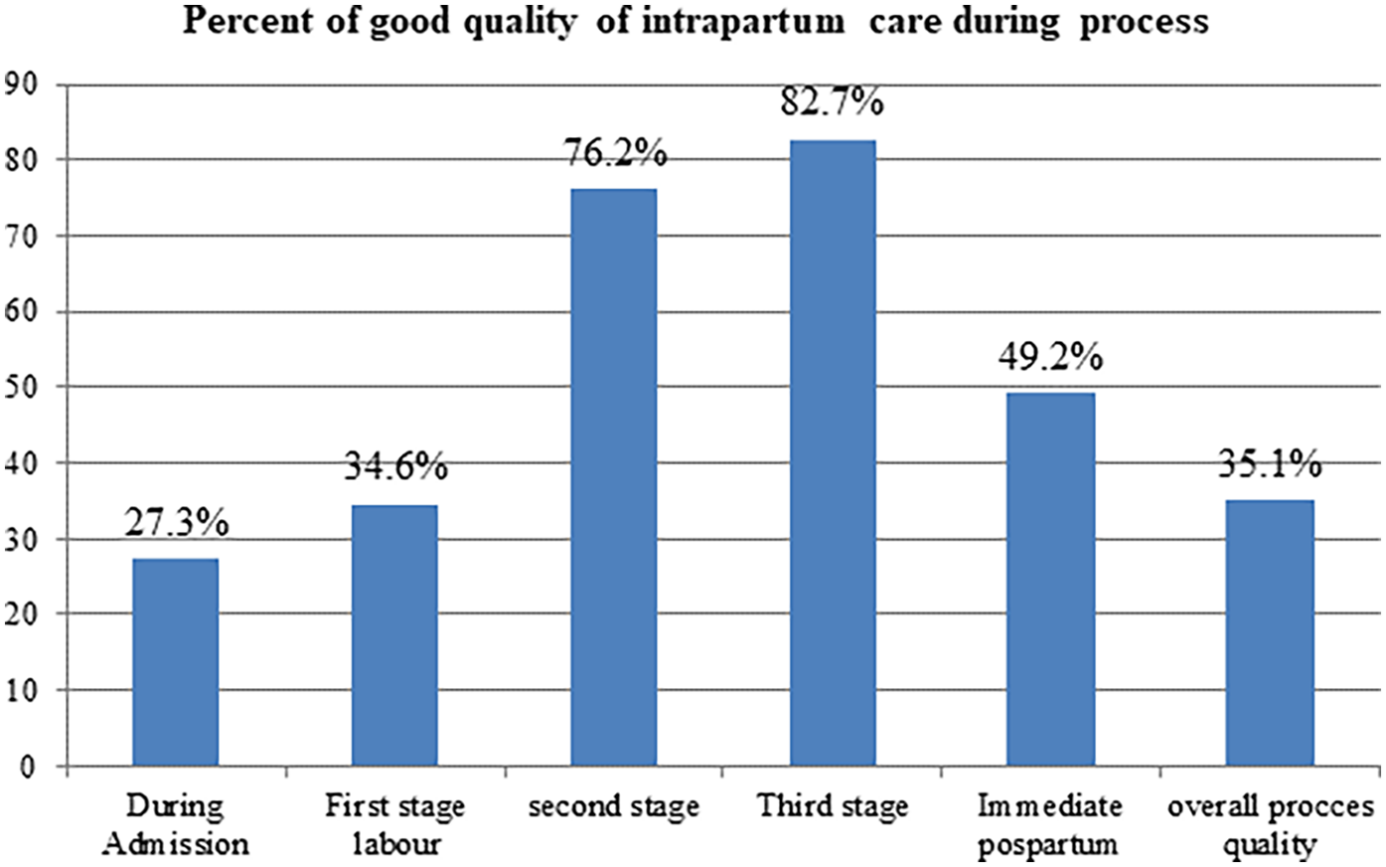
Proportion of good quality of intrapartum care in public health facilities of Wolkite town, Central Ethiopia, 2023.

In this study, 185 deliveries were observed, and only 35.1% (95% CI: 28.3%, 41.9%) of mothers received good-quality intrapartum care. During labor admission, first stage, second stage, and third stage of labor and during the immediate postpartum period, 51 (27.3%), 64 (34.6%), 141 (76.2%), 153 (82.7%), and 91 (49.2%) of mothers received good-quality care, respectively (Figure 1). In the current study, 107 (57.8%; 95% CI: 50.4%, 65%) of mothers and newborns received maternal- and newborn-friendly care during the intrapartum period. Additionally, the finding of this study revealed that only 73 (39.5%; 95% CI: 32.4%, 46.9%) laboring mothers' partographs were completely and properly recorded during the progress of labor (Table 3).

**Table 3:** Quality of intrapartum care aspect in public health facilities of Wolkite town, Central Ethiopia, 2023 (*N* = 185).

Indicators	Frequency (%)	
	Yes (%)	No (%)
First stage of labor		
Takes the mother’s temperature	45 (24.3)	140 (75.7)
Takes the mother’s pulse	83 (44.9)	102 (55.1)
Measures the mother’s blood pressure	140 (75.7)	45 (24.3)
Determines the mother’s respiratory rate	55 (29.7)	130 (70.3)
Measures the mother’s fundal height	133 (71.9)	52 (28.1)
Evaluates uterine contractions (frequency and duration over a 10 min period)	137 (74.1)	48 (25.9)
Auscultates fetal heart rate (FHR)	185 (100)	0
Allows a preferred birthing partner	54 (29.2)	131 (70.8)
Adopts the preferred position of the mother during labour	107 (57.8)	78 (42.20
Ensures privacy during labor	75 (40.5)	110 (59.5)
During physical examination		
Puts sterile gloves on both hands	86 (46.5)	99 (53.5
Removes gloves after being immersed in 0.5% chlorine solution and places them in a leak-proof container	0	185 (100)
Records all information on the clinical records	108 (58.4)	77 (41.6)
Starts partograph to follow the progress of labor	121 (65.4)	64 (34.6)
Infection prevention practice		
Cleanses the vulva with an antiseptic solution before performing vaginal examination	0	185 (100)
Performs limited vaginal examination (e.g., every four hours or indicated)	119 (64.3)	66 (35.9)
Uses sterile gloves when performing vaginal examination or when in contact with body fluids	126 (68.1)	59 (31.9)
Rupture of membranes is not performed routinely	132 (71.4)	53 (28.6)
Preparation to assist the birth		
Prior to delivery, washes hands with running water and soap for 10–15 s and dries an individual clean towel or allows hands to air dry	31 (16.8)	154 (83.2)
Puts sterile double gloves on hands	151 (81.6)	34 (18.4)
At least once encourages the mothers to walk around	142 (76.8)	43 (23.1)
At least once encourages the mothers to change position according to their desire and comfort	107 (57.80)	78 (42.2)
At least once encourages the mothers to take light food or drink fluid in labour	137 (74.1)	48 (25.9)
Allows the mothers to have her preference companion in the labor room	91 (49.2)	99 (50.8)
Gives emotional support to mothers during labor and delivery	144 (77.8)	41 (22.2)
At least once encourages mothers to empty their bladders	134 (72.4)	51 (27.6)
Immediate postpartum period		
Makes sure that the mothers are comfortable (clean, hydrated, and warmly covered)	60 (32.4)	125 (67.6)
Monitors vaginal bleeding	177 (95.7)	8 (4.3)
Bladder distension	89 (48.1)	96 (51.9)
Monitors uterine contraction	126 (68.10	59 (31.9)
Monitors pulse	77 (41.6)	108 (58.4)
Monitors consciousness	119 (64.3)	66 (35.7)
Assists mothers with breastfeeding	82 (44.3)	103 (55.7)
Records the information on the women's clinical record and reports any abnormalities	121 (65.4)	64 (34.6)

### Quality of newborn care

**Figure 2:**
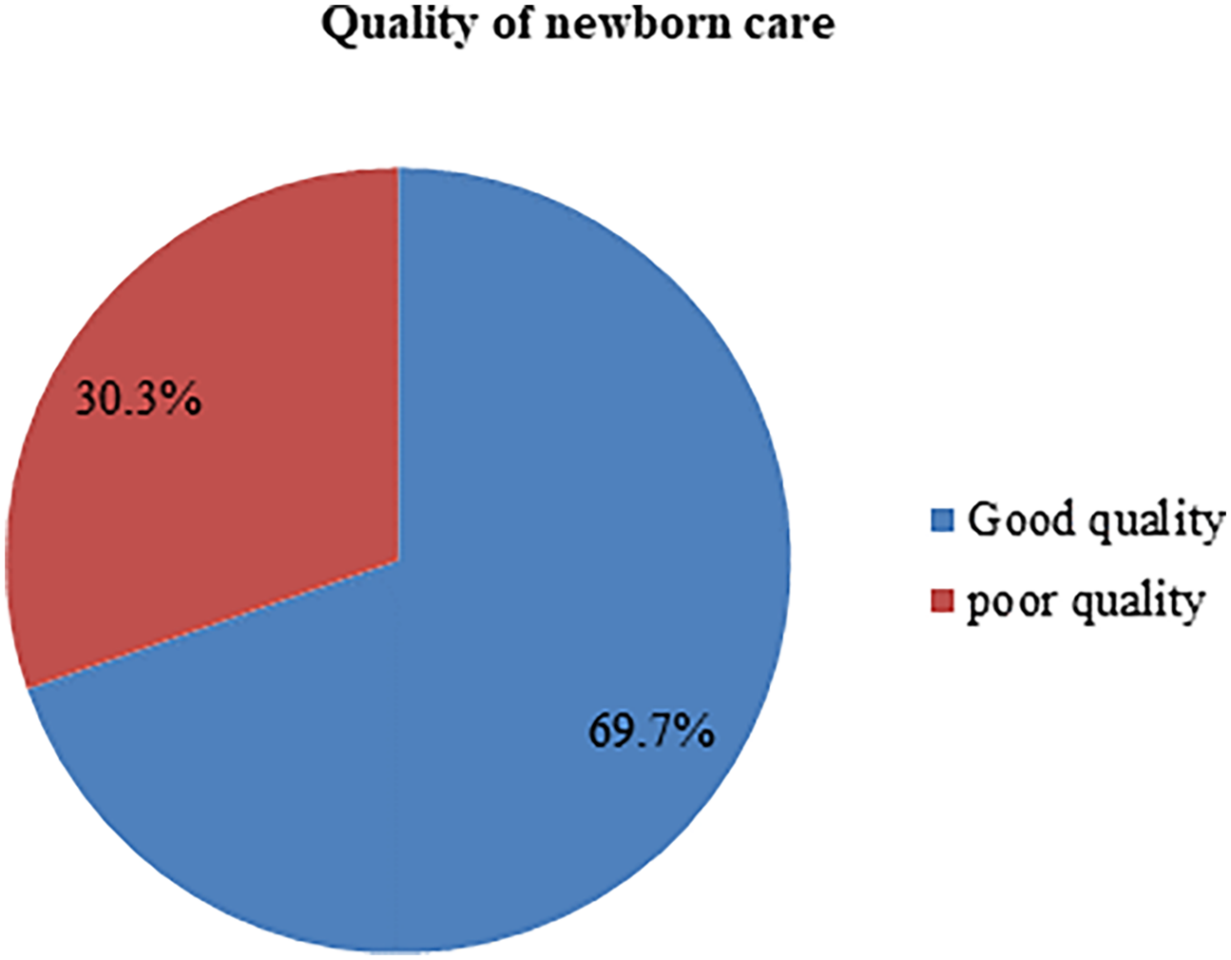
Quality of newborn care in public health facilities of Wolkite town, Central Ethiopia, 2023.

According to the 2016 WHO quality standards, the experience of newborn care was assessed. The findings of this study show that the overall quality of newborn care was 69.7% with 95% CI (62.6%, 76.3%) (Figure 2). In this study, almost all 172 (93%) of newborns started breastfeed within one 1 h. Vitamin K and TTC were administered to 164 (88.6%) and 174 (94.1%) of newborns, respectively. Additionally, only 71 (38.4%) of the newborns were passed to the mothers for skin-to-skin contact.

#### Factors associated with quality of intrapartum care

By using both bivariate and multivariable logistic regression analysis, this study identified that input quality, experience of healthcare providers, training, and maternal- and newborn-friendly care were significantly associated with the quality of intrapartum care.

The odds of mothers who gave birth in health facilities with good quality in input component were 4.5 times more likely to get good-quality intrapartum care than those who gave birth in health facilities with poor input component (AOR = 4. 52 95% CI: 1.31, 14.98). Mothers who get care from health professionals with ≥5 years of experience were seven times more likely to get good overall quality intrapartum care than their counterparts (AOR = 7.23, 95% CI: 1.49, 35.84). Mothers who get friendly healthcare were seven times more likely to receive good overall quality intrapartum care than their counterparts (AOR = 6.89, 95% CI: 1.34, 35.62). In addition, mothers who received care from trained skilled birth attendants were six times more likely to get good overall quality intrapartum care than mothers who received care from professionals who did not get refresher training on intrapartum care (AOR = 5.82, 95% CI: 1.91, 13.61) (Table 4).

**Table 4:** Binary and multilevel logistic regression model of factors associated with good-quality intrapartum care in public health facilities Wolkite town, Central Ethiopia, 2023 (*N* = 185).

Variables	Categories	Quality of intrapartum care		COR (95% CI)	AOR (95% CI)	*P*-value
		Good	Poor			
Age	21–25	24	18	2.67 (1.08, 6.58)	1.14 (0.126, 10.27)	0.909
	26–30	28	76	0.74 (0.33, 1.63)	7.11 (1.34, 17.49)	0.073
	≥31	13	26	1	1	
Sex of proffessional	Male	21	58	0.51 (0.27, 0.96)	0.20 (0.04, 1.01)	0. 062
	Female	44	62	1	1	
Proffession categories	Midwife degree	23	46	2.00 (0.67, 6.01)	0.09 (0.01, 1.81)	0.117
	Midwife diploma	7	22	1.27 (0.35, 4.66)	0.07 (0.003, 1.58)	0.093
	Nurse degree	8	14	2.82 (0.83, 9.64)	0.07 (0.003, 1.86)	0.118
	Nurse diploma	6	9	1.33 (0.20, 8.71)	0.03 (0.01, 1.96)	0.097
	HO	7	6	5.00 (3.65, 13.3)	3.02 (0.06, 16.1)	0.587
	Doctor	5	7	2.86 (0.63, 12.2)	0.26 (0.003, 23.3)	0.554
	Student	5	20	1	1	
Experience	<2 years	10	45	1	1	
	2–4 year	16	39	1.85 (0.75, 4.54)	5.85 (0.54, 23.6)	0.147
	≥5 years	39	36	4.88 (2.14, 11.8)	**7.23 (1.49, 35.84)**	**0.021**
Training	No	42	112	1	1	
	Yes	23	8	7.67 (3.18, 18.7)	**5.82 (1.91, 13.61)**	**0.008**
Knowledge	Poor	20	90	6.7 5 (3.46, 13.8)	0.18 (0.01, 2.80)	0.224
	Good	45	30	1	1	
Appropriate use of partograph	No	24	94	0.16 (0.08, 0.32)	0.65 (0.04, 10.11)	0.759
	Yes	41	26	1	1	
Fiendly care	No	18	57	1	1	
	Yes	47	63	2.36 (1.23, 4.53)	**6.90 (1.34, 35.62)**	**0.021**
Maternal education	Unable to read and write	6	14	0.19 (0.04, 0.86)	1.76 (0.09, 35.89)	0.714
	Read and write	5	20	0.19 (0.04, 0.79)	0.168 (0.01, 3.17)	0.234
	Primary (1–8)	25	65	0.29 (0.09, 0.94)	0.13 (0.01, 1.19)	0.70
	Secondary (9–12)	23	13	1.33 (0.38, 4.67)	0.43 (0.04, 4.81)	0.495
	College/university	8	6	1	1	
Maternal resident	Rural	24	63	0.53 (0.29, 0.98)	0.64 (0.18, 2.23)	0.486
	Urban	41	57	1	1	
ANC follow-up for current birth	No	6	11	0.89 (0.13, 1.22)	0.07 (0.03, 1.58)	0.094
	Yes	64	104	1	1	
Input quality	Poor	7	67	1	1	
	Good	62	7	12.11 (8.89, 38.87)	**4.52 (1.31, 14.98)**	**0.000**
Type of health facility	Health center	13	41	2.54 (0.89, 7.77)	1.14 (0.13, 10.27)	0.909
	Primary hospital	47	39	7.83 (3.47, 26.9)	9.87 (1.34, 25.49)	0.076
	Referral hospital	6	39	1	1	

Bold values indicate significantly associated factors.

## Discussion

The finding of this study indicates that some institutions met the requirements to give high-quality care based on the standards of care in one dimension but not in others. This study shows that none of the skilled healthcare professionals have received the capacity building to manage obstetric complication training in all health institutions and that only one (20%) facility skilled birth attendant was trained to manage neonatal resuscitation to provide quality care, which is consistent with the findings of a study conducted in Addis Ababa, where none of the skilled birth attendants had completed all components of Comprehensive Emergency Obstetric and Newborn Care (CEMONC) training (34). The similarity in the governing health system could be a contributing factor.

In this study, three (60%) of the health facilities met the necessary input quality aspect, which was comparable with a study conducted in Bangladesh where only 57.1% and 52.7% of district hospitals and maternity and child welfare centers met all items of infrastructure, respectively (35). The finding of this study is also consistent with that of another study conducted in Zanzibar, where out of five hospitals, only 60% fulfilled the minimum standards of a skilled healthcare provider (36). The argument is based on the fact that all of the above nations are African nations with comparable economic prospects to the region under investigation. This shows that infrastructure is influenced by the economy of the countries. In contrast, a study in Jabi Tehinan district, Ethiopia, indicated that 49% of mothers had access to good-quality human and material resources (37).

In this study, only 40% of the facilities had water accessibility in delivery wards, similar to a study in Nigeria where only 38% of facilities had running water near the delivery ward (38). However, a study in Tanzania and Kenya showed that only 7% and 18% of facilities had water accessibility, respectively (39). In this study, only 40% and 80% of facilities had functional incubators and bags/masks for newborn resuscitation, respectively, similar to a study in Kenya, where only 50% and 80% of the hospitals had incubators and bags/masks for newborn resuscitation (40). Such variation could occur as a result of the system’s strength and the focus on healthcare service. In this study, only two (40%) health facilities provided towels for drying and covering the babies immediately after birth. This finding was in line with another study conducted in southern Ethiopia where 45.6% of mothers dried their babies with prepared towels (41). A study conducted in six sub-Saharan African countries revealed that the largest gap in newborn care was the lack of towels for drying the newborns (42), which is a basic action for preventing hypothermia.

Developing detailed and written standards of care is crucial for good-quality care at the time of childbirth, and the process aspect is actual intrapartum care between provider and client interaction (28). In this study, only 35.1% (95% CI: 28.3%, 41.9%) of mothers received good-quality intrapartum care, which was consistent with a study conducted in the northern part of Ethiopia in which only 31.25% of health facilities met process quality care (28). The similarity in the governing health system, the low levels of health professional's skills, and the comparably poor health infrastructure could be contributing factors. On the other hand, the finding is higher than that in a study in Ghana in which only 18% of health facilities met process quality care (43) and lower than that in a study in Ethiopia in the Wolayta zone in which only 40.8% of mothers received the standard care (44). The incompetence of the healthcare experts, an insufficient quantity of healthcare professionals, and the lack of quality monitoring could be causes of poor-quality intrapartum care (45).

The lack of adherence to recommended infection prevention procedures puts healthcare professionals, clients, and the general public at a greater risk of contracting infection and illness and leads to low-quality care. In this study, infection prevention practices, such as hand washing before physical examination, were performed only for 8.1% of mothers and only for 31 (16.8%) of mothers before delivery. This was similar to a study in Côte d'Ivoire where only 6.71% of caregivers practiced washing their hands before the examination (46). This study showed that proper gloving on both hands before vaginal examination was practiced only for 46.5% of mothers, which is higher than that in a study in Burkina Faso where 7.96% of care providers used sterile gloves for vaginal examinations (46). This could be caused by the different types of facilities including study settings and the lack of skilled care providers.

In this study, proper partograph utilization was 39.5% (95% CI: 32.4%, 46.9%), which was higher than that in various studies conducted in different regions of Ethiopia where 10.1%, 25%, and 23.8% of the facilities used partograph properly (28, 47, 48). This variation could be due to the availability of well-planned and executed programs, effectiveness of mentoring, supportive supervision, and commitment towards using partographs routinely for each laboring mother.

The result of this study showed that only 57.8% (95% CI: 50.4%, 65%) of mothers and newborns experienced friendly care throughout delivery and the immediate postpartum period. This finding is higher than that in a study carried out in Tigray, which revealed that only 47.2% of mothers and newborns experienced friendly care throughout delivery (31). This could have an impact on future prenatal, delivery, and postpartum maternal health service consumption and reduce maternal and infant mortality. The result of this study showed that only 29.2% of mothers were allowed companionship during delivery, which was higher than that in a study in Arbaminch town (13.8%), Ethiopia, and this finding was similar to the findings of a study in Kenya and Bangladesh where only 29% and 39% of mothers were allowed companionship during childbirth, respectively (49–51). The closeness between these findings could be due to the actual quality of care that was delivered during childbirth.

In this study, the overall immediate postnatal care for the women and their newborns before discharge from the health facilities was only 49.2% (95% CI: 42.2%, 56.2%), which was higher than that in a study done in theWolaita zone (34.9%) in Southern Ethiopia (52). The reported discrepancy is related to the scope of the studies, study sites, and the components of quality measurement indices. Since, postpartum hemorrhage is a danger that all newly delivered mothers face, the WHO postnatal guidelines recommend that all postpartum women have a routine screening for vaginal bleeding, uterine contraction, temperature, and pulse rate during the first 24 h (13). In this study, only 68.1% of mothers were checked for uterine contraction. This suggests that care providers are missing procedures, underperforming, or neglecting to provide care for mothers within the immediate postpartum period. However, the finding was higher than that in another study conducted in Tigray region primary hospitals at 45.5% (53). This variation could be due to different study setting and study time. This indicates the need to have regular updating and auditing in the clinical areas to ensure the best standard care practices.

The overall newborn quality of care in this study was 69.7% (95% CI; 62.6%, 76.3%). The finding is almost similar to that of a study in Tigray in that 67.6% of newborns received good-quality newborn care (31). It is higher than that of a study conducted in West Guji which showed that the good-quality essential newborn care was only 17.9% (54). This discrepancy in the outcome could be caused by differences in the measuring criteria used to determine the quality of newborn care, the type of health institutions, the study sample employed, and the study setting.

This study found that 38.4% of newborns were ensured skin-to-skin contact with their mothers, similar to a study in other regions of Ethiopia which showed that only 35% of newborns had skin-to-skin contact with their mothers (11). In contrast, this finding is lower than that in a study conducted in Kenya in which 57% of newborns received skin-to-skin contact with their mothers after childbirth (55). Although they received capacity-building training and are aware of the benefits of skin-to-skin contact, most healthcare professionals have not implemented behavioral changes to comply with the criteria, which could be the cause of this mismatch.

This study found that input quality, training, health professional experience, and maternal- and newborn-friendly care were significant predictors of good-quality intrapartum and newborn care. The findings of this study show that health facilities with good input quality care provided better intrapartum and newborn care. Facilities with good infrastructure and other resources were more likely to provide better quality care than their counterparts (56). Healthcare providers who received training in partographs, basic emergency obstetric and infant care training, and proper training in evidence-based intrapartum care provided better intrapartum and newborn care than their counterparts. This finding is supported by a study conducted in another region of Ethiopia (57).

Additionally, this research showed that care provider's experience were independent predictors of observed intrapartum and newborn care quality. This finding was supported by a study conducted in Wolayta zone, Southern Ethiopia (44). The study indicated that maternal- and newborn-friendly care was also a significant predictor of observed service quality. This finding was in line with a study conducted in Tigray which revealed that mother- and infant-friendly care services was one of the independent predictors of quality intrapartum and newborn care (31, 33).

## Strength and limitation of the study

### Strength of the study

This study aimed to evaluate various aspects of quality, including the availability of basic infrastructure, essential medications and instruments, the competence of providers (process), and predictors of good-quality intrapartum care. Different approaches (interview, service provision observation, and document review) were used for specific purposes. The study included all of the public health facilities in Wolkite town to make them representative of the findings. The use of the two aspects of quality (input and process) and their association was the most advantageous aspect of this study.

### Limitation of the study

A limitation of this study is the possibility of observer bias among data collectors and the Hawthorne effect. However, to reduce this impact, the first three observations from each skilled attendant were excluded. The observation aspect of this study did not include caesarian section deliveries. The majority of laboring women arrived at the facility after the first stage of labor had been completed at home, which necessitated extending the data collection period to recruit enough participants to meet the sample size. Additionally, it is difficult to observe the second stage of lobar when some laboring mothers elect to have a cesarean section. The application of multilevel analysis would have helped identify individual- and group-level factors associated with the quality of intrapartum care.

## Conclusion

The quality of intrapartum and newborn care in this study area was suboptimal. Clients are not receiving intrapartum care with state-of-the-art knowledge and current clinical best practices. Input quality, training, health professional experience, and maternal- and newborn-friendly care were significant predictors of good-quality intrapartum and newborn care. The local and national health systems should focus on improving the infrastructure of public health institutions including the sustainable provision of medicines, basic supplies, diagnostic services, and water and sanitary facilities required for maternal and newborn care. Additionally, regular monitoring of health facilities is crucial to improve the quality of intrapartum and newborn care provided. Care providers' adherence to the standard procedures and guidelines should be improved through training, supervision, and regular monitoring and evaluation. It is also important to implement respectful maternity care to improve intrapartum care by supporting mothers during delivery, ensuring consented care, and preventing abandonment of care. Further studies should be conducted to identify additional factors affecting the utilization of quality intrapartum and newborn care.

## Data Availability

The original contributions presented in the study are included in the article/Supplementary Material; further inquiries can be directed to the corresponding author.

## Supplementary material

Supplementary File S1

SPSS data set for the study of quality of intrapartum and newborn care in public healthcare facilities of Wolkite town, Gurage Zone, Central Ethiopia.

Supplementary File S2

File All items used for the study of quality of intrapartum and newborn care in public healthcare facilities of Wolkite town, Gurage Zone, Central Ethiopia.
